# White Photoluminescent Ti_3_C_2_ MXene Quantum Dots with Two‐Photon Fluorescence

**DOI:** 10.1002/advs.201801470

**Published:** 2019-03-10

**Authors:** Siyu Lu, Laizhi Sui, Yuan Liu, Xue Yong, Guanjun Xiao, Kaijun Yuan, Zhongyi Liu, Baozhong Liu, Bo Zou, Bai Yang

**Affiliations:** ^1^ College of Chemistry and Molecular Engineering Zhengzhou University Zhengzhou 450000 China; ^2^ State Key Lab of Supramolecular Structure and Materials College of Chemistry Jilin University Changchun 130012 China; ^3^ State key Laboratory of Molecular Reaction Dynamics Dalian Institute of Chemical Physics Chinese Academy of Sciences 457 Zhongshan Road Dalian 116023 China; ^4^ State Key Laboratory of Superhard Materials College of Physics Jilin University Changchun 130012 China; ^5^ College of Chemistry and Chemical Engineering Henan Polytechnic University Jiaozuo 454000 China

**Keywords:** MXenes, pressure, Ti_3_C_2_ quantum dots, two‐photon fluorescence, white fluorescence

## Abstract

A recently created class of inorganic 2D materials, MXenes, has become a subject of intensive research. Reducing their dimensionality from 2D to 0D quantum dots (QDs) could result in extremely useful properties and functions. However, this type of research is scarce, and the reported Ti_3_C_2_ MXene QDs (MQDs) have only shown blue fluorescence emission. This work demonstrates a facile, high‐output method for preparing bright white emitting Ti_3_C_2_ MQDs. The resulting product is two layers thick with a lateral dimension of 13.1 nm. Importantly, the as prepared Ti_3_C_2_ MQDs present strong two‐photon white fluorescence. Their fluorescence under high pressure is also investigated and it is found that the white emission is very stable and the pressure makes it possible to change from cool white emission to warm white emission. Hybrid nanocomposites are then fabricated by polymerizing Ti_3_C_2_ MQDs in polydimethylsiloxane (PDMS) solution, and the bright white emitting hybrid materials in white light‐emitting diodes are used. This work provides a facile and general approach to modulate various nanoscale MXene materials, and could further aid the wide development of applications for MXene materials in various optical‐related fields.

## 1 Introduction

A new class of 2D early‐transition‐metal carbides (MXenes) has been synthesized by selectively etching the “A” element from MAX phases, which are layered carbides or nitrides, where M is an early transition metal, A is an A‐group atom, and X is C and/or N.^1^, ^2^, ^3^, ^4^, ^5^, ^6^, ^7^, ^8^ MXenes possess both metallic conductivity and hydrophilicity, and their surfaces are terminated with hydrophilic groups that provide intercalation or surface redox capacitances with high conductivity (2400 S cm^−1^, comparable to multilayer graphene). The outstanding properties of MXenes distinguish them from traditional 2D materials and make them promising candidates for energy storage and environmental applications, such as supercapacitors, lithium‐ion batteries, and pollution treatment.^8^, ^9^, ^10^, ^11^, ^12^, ^13^, ^14^, ^15^, ^16^, ^17^ In addition, Ti_3_C_2_ MXene exhibited a high antibacterial efficiency, with growth inhibition of 97.70% for *Escherichia coli*, and ultrathin MXene‐based field‐effect transistors with high biocompatibility could be utilized to perform highly sensitive label‐free detection of dopamine, revealing that MXenes could serve as novel nanomaterials with great potential in environmental and biomedical applications.^17^, ^18^, ^19^, ^20^, ^21^, ^22^, ^23^, ^24^, ^25^, ^26^, ^27^, ^28^, ^29^, ^30^

The recent decomposition of 2D nanosheets into 0D nanodots has been used to develop materials for photocatalysis, sensors, and energy conversion owing to the unique confinement effect of 0D structures. Carbon dots,^12^ MoS_2_ nanodots, g‐C_3_N_4_ nanodots, and BN nanodots of less than 10 nm in size have shown different physical and chemical properties from their bulk materials due to the combination of quantum confinement, large surface area, and edge effects.^31^, ^32^, ^33^, ^34^, ^35^, ^36^, ^37^ In the same context, reducing the lateral size of MXenes to the nanoscale may further increase their diversity. There are few reports of such nanoscale reductions in Ti_3_C_2_. For example, monolayered Ti_3_C_2_ MXene quantum dots (Ti_3_C_2_ MQDs) of 2.9 nm in size have been produced by a top‐down hydrothermal method.^38^ They show excitation‐dependent photoluminescence with a quantum yield around 10%, which could be useful for biomedical and other optical applications. Geng and co‐authors have demonstrated a general and mild approach for creating ultrasmall MXenes by simultaneous intralayer cutting and interlayer delamination. The obtained Ti_3_C_2_ nanosheet was a lateral dimension of 2–8 nm and exhibited bright blue‐green fluorescence with the center at 507 nm.^39^, ^40^ Functionalized Ti_3_C_2_@PEG quantum dots have also been produced by a top‐down hydrothermal method. They exhibit excitation‐wavelength and pH‐dependent blue photoluminescence, with a quantum yield of 7.13%.^41^ Li and co‐authors reported N‐doped Ti_3_C_2_ MXene quantum dots (N‐MQDs) made via hydrothermal treatment from a Ti_3_C_2_ precursor and an ethylenediamine nitrogen source. The prepared N‐MQDs exhibited an excitation‐dependent photoluminescence (PL) spectrum with quantum yields of up to 18.7% due to strong quantum confinement.^42^ Although photoluminescent Ti_3_C_2_ MQDs have been obtained, only blue emission has been reported. Efficient differentiation of the wavelength or white emissive Ti_3_C_2_ MQDs are scarce due to a lack of effective synthesis methods and the blur of luminescence mechanism, which greatly hinders their development and applications.

Herein, we unprecedentedly designed a facile, high‐output method for the fabrication of Ti_3_C_2_ MQDs with direct white photoluminescence (photoluminescence quantum yield, PLQY, 9.36%) and two‐photon fluorescence (TPFL). The full width at half maximum (FWHM) was over 220 nm, which covers all of the visible region. High‐pressure studies of Ti_3_C_2_ MQDs Ti_3_C_2_ MQDs were also performed, demonstrating a stable white emission that changed from cool white emission to warm white emission. Our work paves a general to achieve nanoscale MXene materials with enhanced applications in various optical‐related fields.

## 2 Results and Discussion

**Figure 1:**
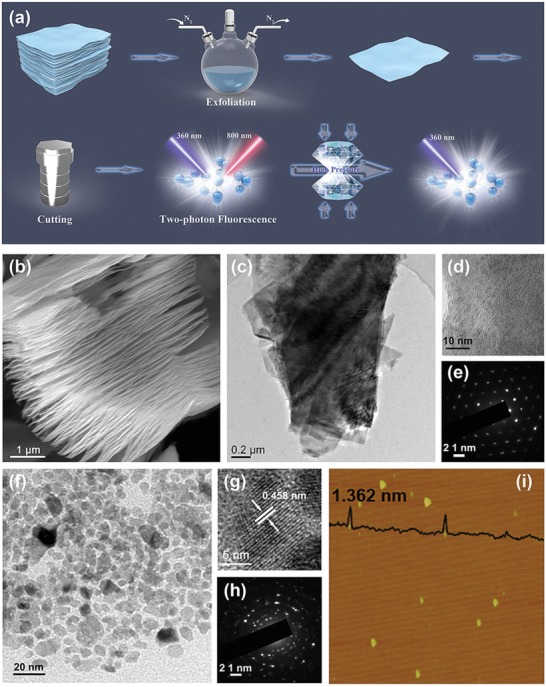
a) Schematic illustration of the preparation and application of Ti_3_C_2_. b) SEM image of bulk Ti_3_C_2_. c) TEM and d) HRTEM images of the Ti_3_C_2_ nanosheet. e) Corresponding SAED patterns of the Ti_3_C_2_ nanosheet in (c). f) TEM and g) HRTEM images of Ti_3_C_2_ MQDs. h) Corresponding SAED patterns of the Ti_3_C_2_ MQDs in (f). i) AFM image of Ti_3_C_2_ MQDs.

The synthesis of Ti_3_C_2_ MQDs is outlined in **Figure** **1**a. Briefly, bulk Ti_3_C_2_ MXene was obtained by selectively etching the Al layer from the Ti_3_AlC_2_ MAX phase with 48% HF acid. Functionalized Ti_3_C_2_ MQDs were further prepared via a two‐step facile solvothermal method using bulk Ti_3_C_2_ as the starting material and oleylamine (OLA) as a surface passivation agent. Typical scanning electron microscope (SEM) and transmission electron microscopy (TEM) images (Figure 1b–d) of the as‐prepared bulk Ti_3_C_2_ and Ti_3_C_2_ nanosheet. Figure 1f show that Ti_3_C_2_ MQDs are successful fabrication of monodispersed MQDs with an average size of 13.1 nm. The MQDs' lattice is clearly resolved in the high‐resolution TEM (HRTEM) image in Figure 1g, showing good crystallinity and a lattice spacing of 0.458 nm, assigned to the (004) planes of Ti_3_C_2_. The clear and sharp selected area electron diffraction (SAED) pattern in Figure 1h also indicates high crystallinity. The HRTEM results suggest that Ti_3_C_2_ MQDs exhibit the pristine structure of MXene due to the low reaction temperature and the protection of surface‐terminated —NH groups under the reducing environment created by the OLA solution. In addition, the MQDs have a hybrid structure with C–Ti in the core and OLA at the surface. Figure 1i shows atomic force microscopy images of the MQDs, providing a relatively precise thickness distribution that indicates an average thicknesses of 1.362 nm, revealing that most of the MQDs include two layers. Energy‐dispersive X‐ray spectroscopy (EDX) further indicated the presence of Ti (60.57%), C (31.50%), and O (7.93%), which implied the formation of Ti_3_C_2_ MQDs (Figure S1, Supporting Information).

**Figure 2:**
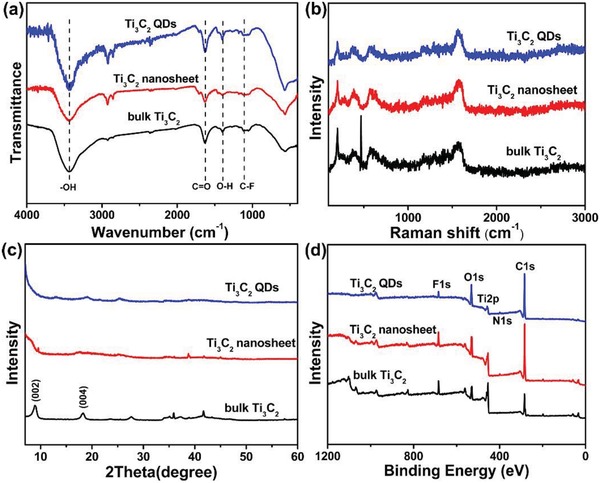
a) FTIR spectra, b) Raman patterns, c) XRD patterns, and d) XPS spectra of bulk Ti_3_C_2_, Ti_3_C_2_ nanosheets, and Ti_3_C_2_ MQDs.

The structure, surface composition, and functional groups of the as‐prepared Ti_3_C_2_ MQDs were verified through Fourier transform infrared (FTIR) spectroscopy and X‐ray photoelectron spectroscopy (XPS). The FTIR spectra of Ti_3_C_2_ nanoparticles exhibit a broad band around 3303 cm^−1^, corresponding to —OH stretching vibration. Another two obvious FTIR bands at 1667 and 1060 cm^−1^ correspond to the stretching vibrations of C=N and C—N (from OLA). The spectra of the Ti_3_C_2_ MQDs show two strong transmission bands at around 2900 and 1500 cm^–1^, which were assigned to the internal vibration of amide bands and the N–H stretching vibration, respectively, indicating that the MQDs were successfully functionalized with OLA. The Raman spectra in **Figure** **2**b for bulk Ti_3_C_2_, Ti_3_C_2_ nanosheets, and Ti_3_C_2_ MQDs are similar in all cases, and all show the Ti–C vibrations, indicating their structural consistency. Figure 2c shows the X‐ray diffraction (XRD) patterns for these three Ti_3_C_2_ materials. Compared with the pattern for the bulk, the XRD patterns for the nanosheets and MQDs are broadened, lower in intensity, and shifted toward lower angles. The XPS results in Figure 2d further demonstrate that the MQDs contained titanium, carbon, nitrogen, fluoride, and oxygen, indicating the presence of —O, —NH, —OH, and —F terminations on the Ti_3_C_2_ surface. Moreover, high‐resolution Ti 2p XPS spectra exhibit four deconvoluted peaks (Figure S2, Supporting Information), corresponding to Ti–O 2p and Ti–C 2p, in agreement with the above HRTEM and EDX results. Overall, the MQDs appear to have been successfully synthesized. While the strong covalent Ti—C bonding was to some extent broken, all of the characterizations suggested that the chemical structure is composed of well‐maintained host layers without any serious damage, demonstrating the reaction efficiency and safety of our method.

**Figure 3:**
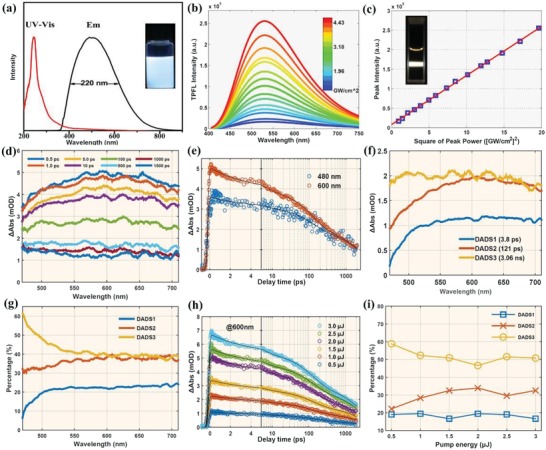
a) UV–vis absorption, fluorescence excitation, and emission spectra (the inset photograph is under UV light). b) Two‐photon spectra with different laser excitation intensities from a 800 nm femtosecond pulse laser (the inset shows a photograph of a solution of Ti_3_C_2_ MQDs with the 800 nm laser passing through). c) Relationship between the two‐photon emission intensity and the square of laser excitation intensity has a linear slope of 1. d) TA spectra of Ti_3_C_2_ MQDs at indicated delay times from 0.5 ps to 1.5 ns. e) Kinetic decay traces at 480 and 600 nm. Black solid lines are fitted curves. f) Results of global fitting with five exponential decay functions showing five decay associated difference spectra (DADS). g) Percentage contributions of the three decay processes to the total dynamic within different wavelengths according to fitted DADS. h) Percentage contributions of the three decay processes to the total dynamic relating to surface state at different excitation energies. i) Percentage contributions of the three decay processes to the total dynamic relating to core state at different excitation energies.

The Ti_3_C_2_ MQDs exhibited strong white luminescence in ethanol solution under UV light. Their strongest emission in ethanol solution was centered at 509 nm with a 220 nm FWHM under 360 nm excitation (**Figure** **3**a). An absolute PLQY of 9.36% was also observed under 360 nm excitation. The unexpected and important TPFL properties of the Ti_3_C_2_ MQDs were investigated using a near‐infrared (NIR) femtosecond (fs) pulsed laser (800 nm). A representative two‐photon luminescence spectrum of the MQDs is shown in Figure 3b, and is similar to the single‐photon fluorescence spectrum. The two‐photon luminescence under pulsed infrared laser excitation was confirmed by comparing excitation under different laser powers. Figure 3c shows a clear quadratic relationship between the excitation laser power and the luminescence intensity, confirming that excitation with two NIR photons was responsible for the white luminescence of the Ti_3_C_2_ MQDs (Figure S3, Supporting Information). Compare to other photoluminescent QDs, perovskite QDs, and inorganic QDs always exhibited much higher PL quantum yields, and narrow, finely tunable emission spectra.^43^, ^44^ As for the white‐emission perovskite QDs, combinations of a self‐trapped excitons associated with the octahedra distortion upon irritation were responsible for the photoluminescence mechanism. They are considered building blocks for various solar cells, light‐emitting diodes (LEDs), and photodetectors.^45^, ^46^ However, Ti_3_C_2_ MQDs have a great impact by ligands with largely of carbon, unique structure and composition. Accordingly, in some instances, they can be made to behave more nearly carbon dots.

To gain further insight into the luminescence mechanism and the carrier relaxation dynamics of the Ti_3_C_2_ MQDs, fs transient absorption (fs‐TA) measurement was carried out under excitation at 400 nm. Figure 3d shows TA spectra of the MQDs excited at 2 µJ with different time delays. There is only a positive band from 480 to 710 nm, which is assigned to excited‐state absorption (ESA). The ESA signal remained almost unchanged during the first 1 ps, and then gradually decreased with increasing delay time. However, the ESA signal only decayed to 20% of the total intensity within the detection time range; this could result in strong fluorescence of the MQDs.^47^, ^48^, ^49^ Kinetic decay traces of two different wavelength are shown in Figure 3e.

**Figure 4:**
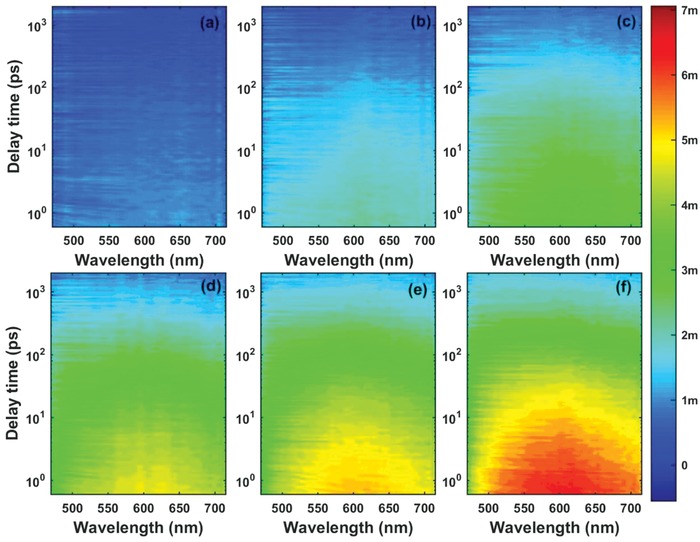
2D pseudocolor maps of transient absorption (TA) spectra expressed in ΔOD as functions of both delay time and probe wavelength for the Ti_3_C_2_ MQDs with a pump wavelength of 400 nm at excitation energies of a) 0.5, b) 1.0, c) 1.5, d) 2.0, e) 2.5, and f) 3.0 µJ.

To explore the detailed relaxation channels and excited state lifetime, we used three exponential decay functions convolved with the instrument response function to globally fit the TA spectra. The fitted lifetimes of excited carriers are 3.83 ± 0.02 ps, 121.9 ± 0.5 ps, and 3.05 ± 0.01 ns. From Figure 3f, we can assess in detail the fitted decay associated difference spectra (DADS) of the Ti_3_C_2_ MQDs. The third DADS has the greatest signal intensity. To evaluate the proportion of the different components in the decay dynamics, the three DADS of different wavelengths are normalized, as shown in Figure 3g. There are two different regions: 1) the percentage of the third component is at a maximum and decreases with increasing wavelength between 480 and 550 nm, and 2) the percentages of the three components are almost constant from 550 to 710 nm. According to previous reports, the ESA signal originated from two states: the surface state OLA groups situated at the edges, and the Ti_3_C_2_ core state. The ESA signal of the surface state with a lower energy level lies at shorter wavelengths near 505 nm, but the signal of the core state occurs at longer wavelengths at 600 nm. These spectral features from two different excited states are shown in Figure 3d. To distinguish the three lifetimes corresponding to different relaxation channels of the carriers, we measured the fs‐TA of the MQDs at different excitation energies from 0.5 to 3.0 µJ (**Figure** **4**). Separate globally fitting of the TA spectra of the MQDs recorded at different excited energies shows that they all have three components within similar lifetimes. Therefore, we combined the six different 2D matrices into an augmented matrix to fit globally with the same fitted parameters using nonlinear least squares. The fitted lifetimes are almost the same as the lifetimes at 2 µJ, being 3.21 ± 0.05 ps, 119.5 ± 0.8 ps, and 3.14 ± 0.05 ns. Figure 3h shows no obvious signs of carrier saturation in the MQDs. We can exclude the influence of Auger recombination. Optical phonon cooling may have contributed to this fast component (3.21 ps).

We previously divided the TA spectra at 550 nm into two parts corresponding to surface and core states. The DADS of both regions are summed to analyze the variation in the proportion of the three components in the decay dynamics with different excitation energies (Figure 3i). The first component's contribution to both the surface state and the core state is about 20%, and it does not vary with energy, in agreement with the previous analysis. For the surface state, the second component's contribution gradually increases with the energy up to 1.5 µJ, above which it remains unchanged. The third percentage shown the opposite trend to the second one, and reached 50%. The different trends might be caused by the increased population of carriers, which increases the chance of nonradiative transitions before the saturation of carriers trapped by the surface state. For the core state, the three percentages show no change with energy: the third is 5% lower than the surface state, whereas the second is 5% higher. Because of its longer lifetime and higher contribution, the third component is attributed to radiative transition by electron–hole recombination. Based on the above analysis, we attribute the three components to their corresponding relaxation channels. After excitation, Coulomb‐induced hot carriers at the core state, and trapped by the surface state, will release their redundant energy via optical phonon scattering (3.21 ps). One part of the cooled carriers will experience nonradiative transition to the ground state within 119.5 ps. The others at the surface and core states will emit mixed broad band fluorescence by the recombination of electrons and holes within 3.05 ns. According to the above TA results, the PL evidently arose from the synergistic effect of the Ti_3_C_2_ core and the surface state (OLA), indicating that the single present particle white fluorescence.^50^, ^51^, ^52^, ^53^, ^54^

**Figure 5:**
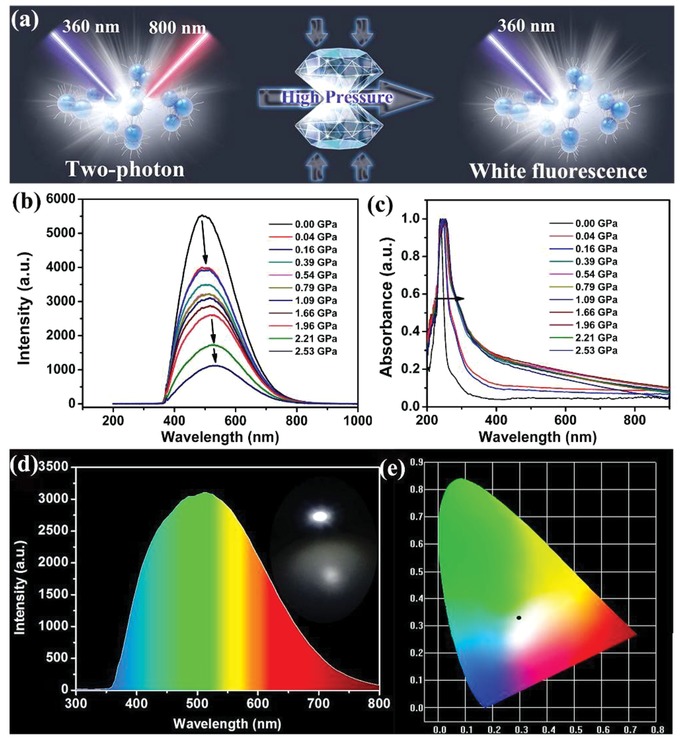
a) Illustration of the preparation and application of Ti3C2 MQDs under high pressure. b) PL spectra of Ti3C2 MQDs with increasing pressure. c) UV–vis spectra of the Ti_3_C_2_ MQDs at selected pressures. d) Photograph (insert) and emission spectrum of the working white LED with pure white emission. e) Color coordinates of the white LED (0.30, 0.34).

To investigate further the piezochromic effect in the Ti_3_C_2_ MQDs, we investigated the influence of applied pressure on their luminescence. Silicone oil was used as a pressure transmitting medium. The actual pressure was determined by monitoring the widths and separation of the R1 and R2 lines of ruby. All in situ high‐pressure experiments shown here were carried out using a symmetric diamond anvil cell (**Figure** **5**a). We employed IIa‐type ultralow fluorescence diamonds with a culet size of 300 mm. A T301 stainless steel gasket was preindented by the diamonds and then drilled to generate a 100 mm diameter cavity for loading the samples. The in situ PL and absorption measurements were performed under high pressure on an Ocean Optics QE65000 spectrometer. The 355 nm line of a violet diode laser and a deuterium halogen lamp were employed as the excitation sources for PL and absorption, respectively. All the experiments were conducted at room temperature. As the applied pressure increased, the fluorescence emission of the Ti_3_C_2_ MQDs clearly showed a gradual red‐shift. Application of pressure up to 2.53 GPa caused a slight color change in the luminescence of Ti_3_C_2_ MQDs, as shown in Figure 5b. However, the MQDs still showed white emission, which made it possible to change from cool white emission to warm white emission with pressure. We carried out further PL experiments by repeatedly exerting and releasing pressure on the MQDs. After two cycles of compression and decompression, the changes in the PL spectra with respect to pressure remained unchanged. This was further evidenced by an irreversible absorption spectrum (Figure 5c), which shows the slightly red‐shifted absorbance width of the MQDs from 0 to 2.53 GPa. Moreover, we investigated the typical pressure‐dependent Raman spectra by utilizing liquid argon was as the pressure transmitting medium to avoid the influence of signal of silicon oil on the Raman spectra. Therein, an Acton SpectraPro 2500i spectrometer equipped with a liquid nitrogen cooled charge‐coupled detector (CCD) camera (Pylon, 100B) was adopted. A 532 nm line from the diode pumped solid state (DPSS) laser with 10 mW power was applied as the excitation source. As shown in Figure S4 (Supporting Information), the Raman spectra evolution of Ti_3_C_2_ MQDs demonstrated that there was nearly no any change of the Raman profile upon increasing pressure to 5.0 GPa, which is in accordance with the stable white emission under high pressure. All of the Ti–C vibration modes of Raman spectra exhibited a negligible shift toward higher wavenumber on pressure, also corroborating the stability of Ti_3_C_2_ MQDs against external pressure stimuli. This phenomenon is attributed to deviation stress resulting from the pressure gradient, given that the OLA chains will crosslink with each other at such a high pressure.^55^, ^56^

The luminescence stability of these Ti_3_C_2_ MQDs was next examined by irradiating them with a UV lamp for 8 h: almost no PL attenuation was observed. The carrier relaxation dynamics of Ti_3_C_2_ MQDs influenced by the surface functional group of OLA will play a crucial role in the PL property, thus the corresponding performance of LED device with white‐emission Ti_3_C_2_ MQDs. We also fabricated white LEDs based on these MQDs to text the potential applications (Figure S5, Supporting Information). We designed hybrid nanocomposites by polymerizing the MQDs in polydimethylsiloxane (PDMS) solution. The PDMS/Ti_3_C_2_ MQDs composites also exhibited white emission, and the FWHM remained over 220 nm. The devices were made by coating PDMS/Ti_3_C_2_ MQDs onto a prototype solid‐state lighting unit consisting of a 365 nm excitation light‐emitting chip, and then polymerizing them in situ.^12^ The emission spectra of these devices (Figure 5d) show emissions ranging from 350 to 750 nm from the white‐emissive PDMS/Ti_3_C_2_ MQDs phosphors. A photograph of the working white LED with pure white emission is also shown. The emission spectra of the MQDs in the LEDs show an emission with color coordinates of (0.30, 0.34) (Figure 5e). We concluded that the white‐emissive PDMS/Ti_3_C_2_ MQDs phosphors are promising candidates for practical lighting applications.

## 3 Conclusion

In summary, a facile, high‐output method for preparing white‐emitting Ti_3_C_2_ MQDs with TPFL was developed. The FWHM was over 220 nm (with a PLQY as high as ≈9.36%), which covers all of the visible region. The TA results demonstrated the carbon core, and the surface state linked with OLA chains synergistically contributed to the white PL. We also examined the piezochromic effect of the fluorescence in the Ti_3_C_2_ MQDs, revealing that the white emission of these Ti_3_C_2_ MQDs is very stable under high pressure and the pressure make it possible to change from cool white emission to warm white emission. Furthermore, we demonstrated white LEDs based on our Ti_3_C_2_ MQDs. We are currently performing further studies on the applications of the TPFL of these Ti_3_C_2_ MQDs.

## Conflict of Interest

The authors declare no conflict of interest.

## Supporting information

Supplementary
