# Supplementary material for: White Photoluminescent Ti3C2 MXene Quantum Dots with Two‐Photon Fluorescence
Source: Adv Sci (Weinh). 2019 Mar 10;6(9):1801470. doi: 10.1002/advs.201801470 (PMC6498120; doi:10.1002/advs.201801470)
Supplement: Supplementary file 1 — Supplementary [file ADVS-6-1801470-s001.pdf]

Copyright WILEY-VCH Verlag GmbH & Co. KGaA, 69469 Weinheim, Germany, 2019.

ADVANCED SCIENCE
Open Access

Supporting Information

for *Adv. Sci.,* DOI: 10.1002/advs.201801470

White Photoluminescent Ti_3C_2 MXene Quantum Dots with Two-Photon Fluorescence

Siyu Lu, Laizhi Sui, Yuan Liu, Xue Yong, Guanjun Xiao,* Kaijun Yuan, Zhongyi Liu, Baozhong Liu,* Bo Zou, and Bai Yang*

White Photoluminescent Ti_3C_2 MXene Quantum Dots with Two-Photon Fluorescence

Siyu Lu*, Laizhi Sui, Yuan Liu, Xue Yong, Guanjun Xiao*, Kaijun Yuan, Zhongyi Liu, Baozhong Liu*, Bo Zou, and Bai Yang

Dr. X. Yong, Dr. Y. Liu, Prof. S. Lu, Prof. Z. Liu

College of Chemistry and Molecular Engineering, Zhengzhou University, Zhengzhou, 450000, China

E-mail: sylu2013@zzu.edu.cn;

Dr. L. Sui, Prof. K. Yuan

State key Laboratory of Molecular Reaction Dynamics, Dalian Institute of Chemical Physics, Chinese Academy of Sciences, 457 Zhongshan Road, Dalian 116023, China

Prof. S. Lu, Prof. B. Yang

State Key Lab of Supramolecular Structure and Materials College of Chemistry, Jilin University, Changchun, 130012, China

Dr. G. Xiao, Prof. B. Zou

State Key Laboratory of Superhard Materials, College of Physics, Jilin University, Changchun 130012, China

E-mail: xguanjun@jlu.edu.cn

Prof. B. Liu

College of Chemistry and Chemical Engineering, Henan Polytechnic University, Jiaozuo 454000, China

Email: b_z_liu@163.com

METHODS

Femtosecond transient absorption setup:

A regeneratively amplified Ti:sapphire laser system (Coherent Libra, 50fs, 1kHz) provides the fundamental light source. The pump pulse (400 nm) is generated by focusing a portion of fundamental light into BBO crystal. In order to avoid the

influence of rotational relaxation effects on dynamics, the polarization of pump pulse is randomized by depolarizing plate. The other fundamental pulse provides broadband probe pulse (white light continuum) that is produced by focusing 800 nm fundamental light into sapphire plate (3mm). The pump and probe beams are overlapped in the sample with crossing areas of 600 μm and 150 μm. After passing through the sample, the probe pulse is focused into optical fiber that is coupled to spectrometer(AvaSpec-1650F). The energy of 400 nm excitation pulse is adjusted to about 1.5μJ/pulse by a neutral density optical filter. The pump pulse is chopped at 500 Hz to acquire pumped (signal) and un-pumped (reference) probe spectra, and the ΔOD spectrum can be obtained by processing them. The solutions are placed in 2 mm optical path length quartz cuvette. Both the instrument response function (100 fs) and temporal chirp in the probe light are determined by measuring the cross modulation of ethanol. The group velocity dispersion effect on the experiment data is corrected by home-made chirp program. For each measurement, the pump-probe delay scan is repeated three times to give the averaged experiment data.

Two -photon fluorescence (TPF) setup:

The excited source consists of a regeneratively amplified Ti:sapphire laser system (Coherent Libra, 50fs, 1kHz, 800 nm). The TPF signal is collected at the right angles from excited solution placed in the 1cm fluorescence cuvette. The signal is coupled into the spectrometer (Spectra Pro 500i,PI Acton) through a fiber. Finally, the TPF signal is detected by an intensified charge coupled device (ICCD, PIMAX4, Princeton Instruments) triggered by laser.

Ti_3C_2Tx suspension:

The MAX phase was prepared by mixing with TiH_2, Al and graphite in a molar ratio of 3:1.1:2, and followed by ball milling for 12 h. The mixture was then heated under a protection of argon (Ar) gas in a tube furnace for 2 h at 1400 °C. The resultant bulk was powdered and sieved by a 400 mesh sieve. The Ti_3AlC_2 powder was soaked into 49% HF solution at 60°C, and then placed in a 70°C vacuum oven overnight to obtain Ti_3C_2Tx powder.

Synthesis of Ti_3C_2 nanosheet:

Ti_3C_2 nanosheets were synthesized in the following method. Briefly, 20 mL oleylamine was added to a 50 mL three-neck flask and heated to 100°C to eliminate air by bubbling nitrogen. 0.01g Ti_3C_2 was added to the solution and the stirring speed adjusted to 500 rpm, and then ventilated with inert gas for 30 min to purge any air. The resulting solution was heated to 250°C and kept at this temperature for 3h.

Synthesis of Ti_3C_2 MQDs:

The Ti_3C_2 MQDs were synthesized by hydrolyzing the as prepared Ti_3C_2 nanosheets. The Ti_3C_2 nanosheets in oleylamine was strongly sonicated for 6 h (600 w), then the uniformly-dispersed solution was added to a Teflon-lined autoclave and heated at 80 °C for 48 h.

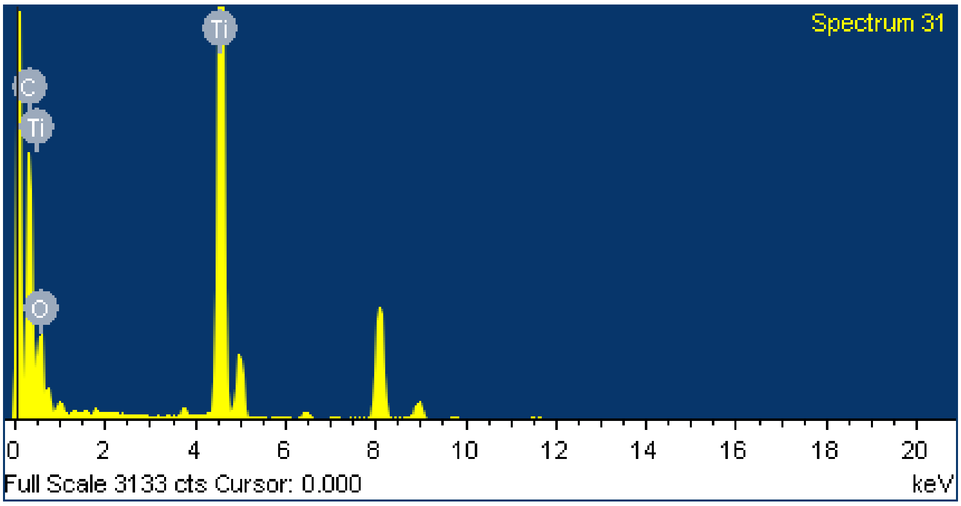

Figure S1. Energy-dispersive X-ray spectroscopy (EDX) of Ti_3C_2 MQDs displaying that the intrinsic chemical structure of Ti_3C_2 was well maintained

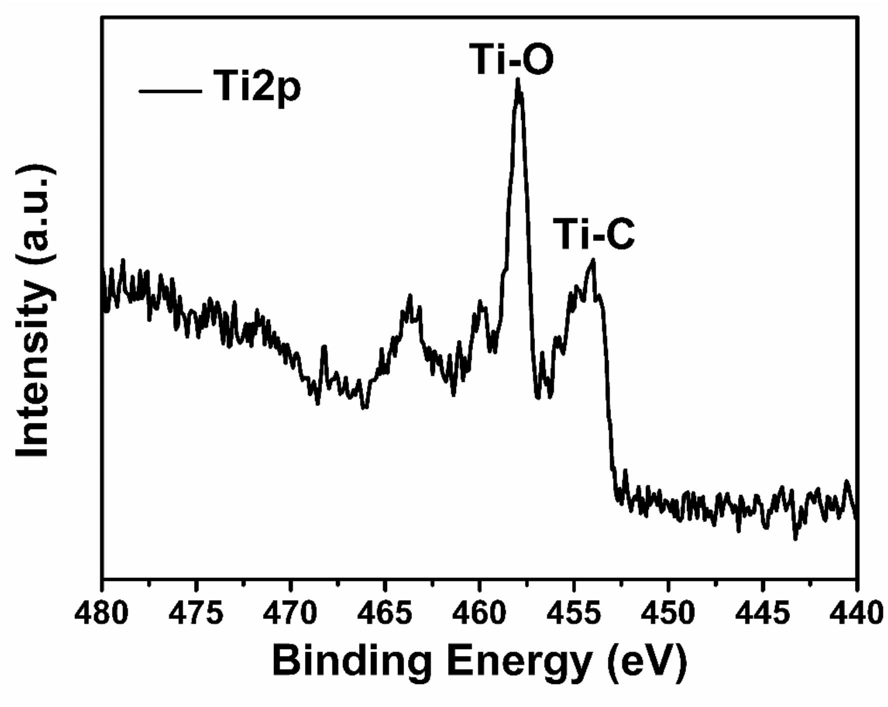

Figure S2. High-resolution XPS spectra of Ti 2p of Ti_3C_2 MQDs displaying that the intrinsic chemical structure of Ti_3C_2 was well maintained.

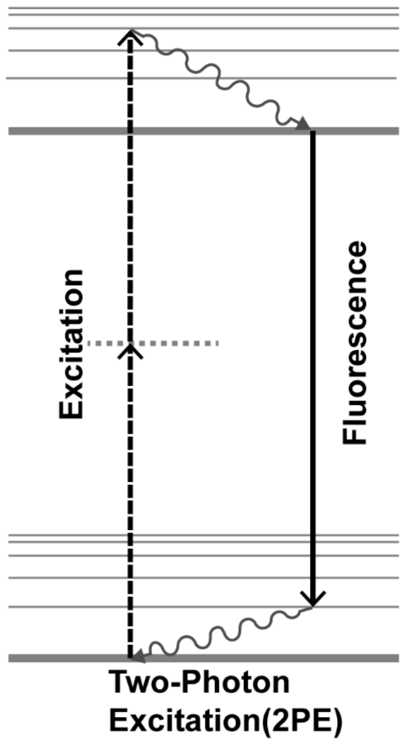

Figure S3. Illustration of the possible mechanism emission process of the Ti_3C_2 MQDs composite.

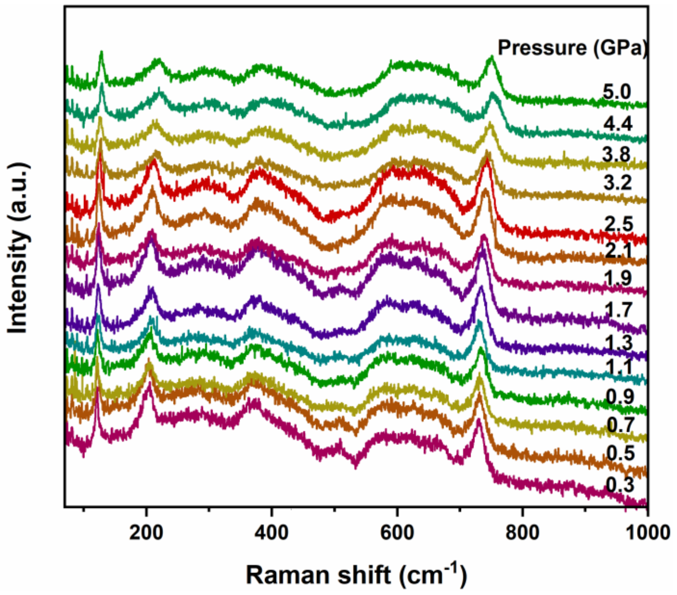

Figure S4. Typical pressure-dependent Raman spectra of the Ti_3C_2 MQDs with liquid argon as the pressure transmitting medium.

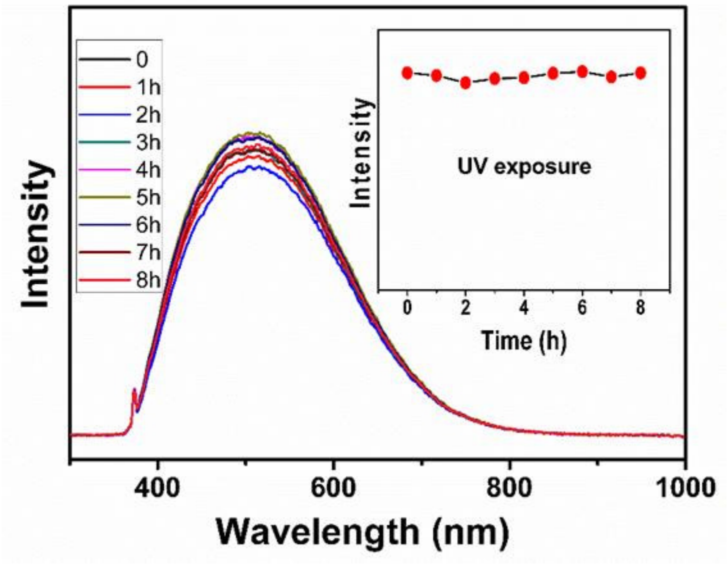

Figure S5. The stability test of fluorescence intensity on excitation time for Ti_3C_2 MQDs in DI water (365nm lamp).
